# Midwifery in Bristol

**Published:** 1940

**Authors:** H. J. Drew Smythe, H. L. Shepherd

**Affiliations:** Consultant Obstetrician, Obstetrical Unit, Southmead Hospital; Consultant Obstetrician, Obstetrical Unit, Southmead Hospital


### MIDWIFERY IN BRISTOL.

BY

H. J. Drew Smythe, M.D., M.S., F.R.C.S., F.R.C.O.G.,

AND

H. L. Shepherd, M.B., Ch.M., F.R.C.O.G.,

*Consultant Obstetricians, Obstetrical Unit, Southmead Hospital.*

---

The Maternity Department was first started at the Bristol General Hospital when in 1854 Dr. Joseph Swayne was appointed Physician Accoucheur. The Bristol Royal Infirmary had no special department for midwifery until many years later when Dr. Wedmore was appointed Obstetric Physician in 1887. The departments developed steadily under Aust Lawrence, Newnham and Rayner at the Hospital and under Walter Swayne and Statham at the Infirmary, until in 1936 the Extern and Intern Departments had grown to such a size that they delivered more than two thousand cases, or a third of the total number for Bristol. Thus these two voluntary institutions provided the main maternity services for B istol and its neighbourhood, and more than one hundred thousand Bristolians must have taken their first breath under their auspices.

However, on 31st December, 1939, the Bristol Royal Infirmary and Bristol General Hospital, recently united as the Bristol Royal Hospital, ceased to undertake the care of maternity cases.

The financial outlay involved in such a service as they had given was very large and the ratepayers of Bristol have been saved many hundreds of thousands of pounds by the work of the voluntary institutions. The Voluntary Hospital Authorities realized that in the present state of financial uncertainty, it would be necessary to decrease their expenditure, and it seemed wiser to give up one department entirely rather than to cut down the work of all departments. If one department had to go, then the Maternity Department was the obvious selection, for Parliament has recognized that the birth of the next generation is not a matter for private charity. Furthermore, there were distinct advantages from the medical and nursing point of view in establishing a single midwifery service in Bristol, with a separate hospital.

The medical staff of the voluntary hospitals, while regretting such a step, realized its neces ity and expressed the opinion that with the co-operation of the Health Committee it might be possible to form one midwifery service based on a new maternity hospital built on the site of the present one in Southwell Street, Kingsdown, the existing buildings of which were not adaptable to conversion into a modern maternity hospital. The Health Committee, however, after consultation with the Bristol Hospitals' Council, undertook the construction of the maternity unit at Southmead because they thought it would be more reasonable to develop this as the main maternity hospital for Bristol. They had in mind the special recommendation of the Departmental Committee on Maternal Mortality, whose report, published in 1937, expressed the opinion that maternity accommodation should be associated, wherever practicable, with a general hospital, where facilities for diagnosis and treatment are readily available.

The decision to give up maternity work at the two voluntary hospitals appeared to be a very serious one, as these hospitals were concerned not only with the care of Bristol cases but the reception of abnormal midwifery from the surrounding counties and also the teaching of midwifery both to medical students and pupil midwives. The teaching of medical students is of the highest importance, since adequate provision for the practice of midwifery is essential to the existence of a Medical School. The Health Committee, wisely recognizing the importance of these problems, give every facility for consultations on difficult cases at their ante-natal clinics and admit such cases to Southmead Hospital. They have also permitted the residence of medical students at the Hospital, in order to attend cases there and domiciliary cases over a large area of Bristol.

Thus, this re-organization has turned out to be a geographical transfer of patients to modern buildings and the unification of the maternity services of Bristol.

The Bristol City Council approached the Obstetric Department of the University of Bristol to provide a consultative service for the municipal hospitals and this was agreed to, so that the present honorary staff of the Gynæcological Department of the Royal Hospital is responsible for the maternity service at Southmead, both for the patients and for the training of medical students. The training of pupil midwives is shared between Southmead and the Bristol Maternity Hospital, and a district has been allotted to each institution by the Health Committee.

The arrangement of the Obstetric unit at Southmead is as follows: The unit provides 100 midwifery beds, 88 for "clean" cases and 12 in a separate block for "suspect" cases, 4 beds of which are completely separate for cases of puerperal sepsis arising in the unit. It is, however, the policy wherever possible to admit and treat cases of puerperal pyrexia at the Isolation Hospital, where the services of the same obstetric consultants are available. The 88 beds are arranged in two similar blocks of 44 beds. Each block consists of a main block of labour wards, operating theatre, sterilizing and reception rooms and two wings lead off from this block each having 22 beds. The rooms contain either one or two beds and there is only one room in either wing which contains more, namely three. This three-bedded room was intended for ante-natal cases, but owing to lack of beds at the present time it has had to be used for labour cases. A ward in the main building is now used for ante-natal cases and over-flow labour cases, and a labour theatre has been provided for this ward.

Each block of 22 beds is in charge of a consulting obstetrician who is called on in any emergency and who pays regular visits for clinical and teaching purposes. An Obstetric Tutor lives at the hospital and is in charge of the whole unit, as Senior Resident Officer under the general administration of the Medical Superintendent. The Tutor assists in the teaching of the students and is available for emergencies or advice on cases in the unit or on the district. The Tutor is in charge of the district. There are two resident obstetric officers each in charge of a block of 44 beds under the supervision of the Tutor. These residents hold the appointment for six months and by this means are able to satisfy the regulations of the Royal College of Obstetricians for Membership and Diploma examinations.

The Consultant Obstetricians hold consultant ante-natal clinics at Southmead Hospital and in other ante-natal centres in the city and so link up the obstetric unit with the municipal ante-natal clinics.

Permission has been obtained from the Health Committee for the admission of abnormal midwifery cases from the adjacent counties and a regional scheme for midwifery is now operating in the area. The counties of Somerset and Gloucester are prepared to take financial responsibility for maternity cases admitted to Southmead which are of an urgent nature on account of medical or other reasons, and the Bristol Health Committee have agreed to allow admission of cases which may present obstetric difficulties at confinement.

The whole unit is under the supervision of the Professor of Obstetrics of the University of Bristol, as Director of Obstetrics, and so forms part of the Department of Obstetrics of the University. Medical students do both their intern and district cases from the Hospital under the supervision of the Obstetric Registrar, residing in the Hospital during a period of three months. Nine students live in at a time, six doing intern cases and three on the district. Pupil midwives are trained for the first part of the C.M.B. Certificate at Southmead Hospital and then go to the Bristol Maternity Hospital for the second part. At each institution a Sister Tutor has been appointed for the purpose of teaching pupil midwives.

The obstetric block at Southmead is an ideal modern obstetrical hospital providing facilities for both normal and abnormal midwifery, including operative midwifery. The small wards increase the patient's comfort and should sepsis occur, prevent its spread to a large number of patients. On the other hand, supervision by the nursing staff is more difficult and an increase in the number of nurses has been found necessary.

The arrangement and equipment of labour and operating theatres is excellent. It consists of reception room, two labour theatres for each 22 beds and an operating theatre for each 44 beds. The only extra accommodation which would have been advantageous is the provision of a room for patients in the first stage of labour, before being taken to the labour theatre.

The present accommodation at Southmead Hospital was not designed to meet the whole needs of Bristol and the adjacent counties, and a further expansion is necessary. The Bristol Maternity Hospital at the present time provides some additional and indispensable accommodation for a large number of patients, and a doctor in general practice may admit cases there where they can be under that practitioner's care. This hospital trains pupil midwives for Part II of the C.M.B. Certificate in its wards and on its district. A second hospital for Part II training is necessary as the Central Midwives Board rule that Part I and Part II training must be carried out at different institutions so that without the Maternity Hospital pupil midwives would have to go elsewhere for this part of their training.

Thus Bristol has the nucleus of a maternity service which links together the University, the Health Department and its medical officers, and the municipal and voluntary hospitals. Its complete development awaits more peaceful times.

